# Could Hyaluronic acid (HA) reduce Bacillus Calmette-Guérin (BCG) local side effects? Results of a pilot study

**DOI:** 10.1186/1471-2490-14-64

**Published:** 2014-08-13

**Authors:** Luca Topazio, Roberto Miano, Valentina Maurelli, Gabriele Gaziev, Mauro Gacci, Valerio Iacovelli, Enrico Finazzi-Agrò

**Affiliations:** 1 School of Specialization in Urology, Tor Vergata University, Rome, Italy; 2 Department of Experimental Medicine and Surgery, Tor Vergata University, Rome, Italy; 3 Department of Urology, University of Florence, Florence, Italy; 4 Unit for Functional Urology, Department of Urology, Policlinico Tor Vergata, Rome, Italy

**Keywords:** BCG, Hyaluronic acid, Non-muscle invasive bladder cancer, Non bacterial cystitis

## Abstract

**Background:**

Bacillus Calmette-Guérin (BCG) is considered the most effective treatment to reduce recurrence and progression of non-muscle invasive bladder cancer (NMIBC) but can induce local side effects leading to treatment discontinuation or interruption. Aim of this exploratory study is to investigate if the sequential administration of Hyaluronic acid (HA) may reduce local side effects of BCG.

**Methods:**

30 consecutive subjects undergoing BCG intravesical administration for high risk NMIBC were randomized to receive BCG only (Group A) or BCG and HA (Group B). A 1 to 10 Visual Analog Scale (VAS) for bladder pain, International Prostate Symptom Score (IPSS) and number of micturitions per day were evaluated in the two groups before and after six weekly BCG instillations. Patients were also evaluated at 3 and 6 months by means of cystostopy and urine cytology.

**Results:**

One out of 30 (3,3%) patients in group A dropped out from the protocol, for local side effects. Mean VAS for pain was significantly lower in group B after BCG treatment (4.2 vs. 5.8, p = 0.04). Post vs. pre treatment differences in VAS for pain, IPSS and number of daily micturitions were all significantly lower in group B. Three patients in group A and 4 in group B presented with recurrent pathology at 6 month follow up.

**Conclusions:**

These preliminary data suggest a possible role of HA in reducing BCG local side effects and could be used to design larger randomized controlled trials, assessing safety and efficacy of sequential BCG and HA administration.

**Trial registration:**

NCT02207608 (ClinicalTrials.gov) 01/08/2014

Policlinico Tor Vergata Ethics Committee, resolution n 69–2011.

## Background

Bacillus Calmette–Guérin (BCG) is considered the most effective treatment to increase disease-free interval and reduce progression of non-muscle invasive bladder cancer (NMIBC) [1]. Although considered safe, BCG can produce both local and systemic side effects leading to treatment discontinuation or interruption. The most common local side-effects of BCG intravesical instillations include cystitis, characterized by irritative voiding symptoms and hematuria, which occur in approximately 75% of all patients. More rarely, serious local adverse events as a result of BCG infection, such as symptomatic granulomatous prostatitis and epididymo-orchitis, might occur and require permanent discontinuation of BCG treatment. Systemic side-effects include flu-like symptoms, such as general malaise and fever, occuring in approximately 40% of patients. A high persistent fever might be related to BCG infection or sepsis. Local and systemic side-effects might lead to discontinue intravesical BCG treatment in approximately 20% of patients [2]. Up to 54% of the patients undergoing intravesical therapy with chemotherapeutic agents to treat superficial bladder tumours can be affected by nonbacterial cystitis [3].

Several solutions have been proposed to reduce the occurrence of side effects from BCG with the aim to limit BCG discontinuation and the concomitant discomfort during endovesical treatment. Some Authors have proposed to avoid BCG administration in case of TUR within previous 2 weeks, traumatic catheterization, macroscopic hematuria, urethral stenosis, active tuberculosis, prior Bacillus Calmette–Guérin sepsis, immuno-suppression or urinary tract infection [4]. Other procedures include the prophylactic administration of isoniazid [5] or ofloxacin [6,7] or usually involve BCG dose reductions [8]. In common practice antimicrobials, anticholinergics, anaesthetics and analgesics are often used to relieve patients’ symptoms.

Glycosaminoglycan (GAG) substitution therapy is an emerging treatment of Bladder Pain Syndrome/Interstitial Cystitis (BPS/IC) and response rates between 30% and 80% have been described with intravesical administration of various GAGs (hyaluronic acid, pentosan polysulfate, heparin, chondroitin sulfate, and dimethyl sulfoxide) [9,10]. Few papers report the results of GAG substitution therapy in the treatment of radiation and chemical cystitis [9,10]. To our knowledge, to date, only two papers have described GAG use in the treatment of BCG local side effects; this papers show very good results, with significant reduction of lower urinary tract symptoms after intravesical administration of HA [11,12].

Aim of the present randomized pilot study was to evaluate if the sequential administration of HA and BCG could be safe in prevention of early recurrence and progression of bladder tumor, and safe in reduction of local side-effects in patients with high risk NMIBC.

## Methods

This is a prospective randomized pilot study approved by our institutional review board (Policlinico Tor Vergata Ethics Committee, resolution n° 69–2011). Patients with a diagnosis of a intermediate/high risk NMIBC, according to the European Organization for Research and Treatment of Cancer (EORTC) score [13] after trans-urethral resection of bladder tumor (TURBT) were eligible for this study.

Inclusion criteria were:

1. Hystologically proven non-muscle invasive bladder cancer;

2. Indication to intravesical instillation of BCG according to EAU guidelines;

3. Age > 18 years;

4. Willingness, to participate to the study;

5. Written informed consent.

Exclusion criteria were:

1. Previous or ongoing BCG or different intravesical instillations;

2. Urinary tract infections (UTI) or other known pathologies of the lower urinary tract;

3. Indication for a radical cystectomy;

4. Severe systemic disorders, including neurological pathologies, kidney, liver or heart failure;

5. Contraindications to BCG use.

Patients enrolled in the study started the intravesical therapies within 4 weeks from the TURBT and were randomized in two groups. We a priori decided to stop this pilot study after reaching the number of 15 patients in each group. Group A was sent to receive BCG (Immucist®81 mg, Sanofi-Aventis Group) alone and Group B to receive BCG (see previous reference) and HA 40 mg (Cystistat, Mylan, Pittsburgh, PA, U.S.A.). BCG strain used in our study was Connaught 3,4 ± 3 × 10(8) CFU, 81 mg (Immucist®) in 50 cc of saline. BCG was administered by means of a 2 ways 14 Ch Foley catheter latex and left inside the bladder for 60 minutes. After 60 minutes BCG was evacuated, the bladder washed with saline and the catheter removed. HA was administered intravesically after every BCG evacuation in Group B. After HA administration and catheter removal, patients were instructed to maintain HA in the bladder as long as possible, for at least 2 hours after administration. In Group A normal saline was administered, so that all patients were blinded to the administration of HA. Both groups received an induction course of 6 weekly instillations of BCG.

A 1 to 10 Visual Analog Scale (VAS) for bladder pain was considered the primary outcome measure of the study and was evaluated in both groups before and after the treatment. International Prostate Symptom Score (IPSS) and number of micturitions per day were also evaluated in both groups before starting and at the end of the induction cycle of BCG instillations.

Patients were evaluated at three and six months by means of cystoscopy and urine cytology for oncologic follow-up. The number of patients with bladder tumor recurrence was recorded in both groups at 6 month follow-up.

Data are presented as mean ± standard deviation (SD). The statistical analysis was conducted with a t Student Test using a software Stata 12.0, and considering as significant a *p value* ≤0.05.

Our manuscript adheres to CONSORT guidelines. CONSORT checklist is shown in Additional file 1.

## Results and discussion

30 subjects were enrolled in the study between January and December 2011. Mean age was 67 (range 54–81): 23 (76%) were male while 7 (24%) female. All Patients had BCa with intermediate/high risk of progression according to the European Organization for Research and Treatment of Cancer (EORTC) score. Fifteen patients (12 male and 3 female) were randomly assigned to Group A and 15 patients (11 male and 4 female) to Group B. Clinical and pathologic features of patients are shown in Table 1.

**Table 1:** Clinical and pathologic features of pts

	**Tot**	**Group A (BCG)**	**Group B (BCG + HA)**
***Gender***			
*M*	23 (76%)	12 (80%)	11 (73%)
*F*	7 (24%)	3 (20%)	4 (27%)
***Age***			
*Mean*	67	66	68
*Range*	54-81	54-75	60-81
***N° of tumors***			
*Single*	9 (30%)	5 (33%)	4 (27%)
*Multiple*	21 (70%)	10 (67%)	11 (53%)
***Tumor diameter***			
*Mean*	1,5 cm	1,5 cm	1,5 cm
*Range*	5 cm – 50 cm	5 cm – 40 cm	5 cm – 50 cm
***pT***			
*Ta*	17 (56%)	9 (60%)	8 (53%)
*T1*	13 (44%)	6 (40%)	7 (47%)
*CIS*	8 (27%)	3 (20%)	5 (33%)
***WHO grade 1973***			
*G1*	8 (27%)	4 (27%)	4 (27%)
*G2*	10 (33%)	6 (40%)	4 (27%)
*G3*	12 (40%)	5 (33%)	7 (46%)
***EORTC recurrence score***			
*≤ 9 intermediate*	10 (33%)	6 (40%)	4 (27%)
*10-17 high*	20 (67%)	9 (60%)	11 (73%)
***EORTC progressive score***			
*≤6 intermediate*	11 (36%)	6 (40%)	5 (33%)
*7-23 high*	19 (64%)	9 (60%)	10 (67%)

Only one out of 30 (3,3%) patients in Group A dropped out from the protocol, for local side effects (urgency, frequency and burning micturition), while all Patients in Group B completed the induction cycle with intravesical BCG. The results are shown in Table 2.

**Table 2:** Results of VAS, IPSS and bladder diaries at baseline and at the end of BCG induction cycle

	**Group A**	**Group B**	**p**	**Group A**	**Group B**	**p**	**Group A**	**Group B**	**p**
***Mean (SD)***	**Pre**	**Pre**		**Post**	**Post**		**Difference**		
*VAS (1–10)*	4.5 (±2)	4.9 (±1.8)	.56	5.8 (±1)	4.2 (±1.6)	.04	1.5 (±0.7)	-0.7 (±1.6)	.0001
*IPSS*	13.9 (±4.4)	14.7 (±4.0)	.60	17.5 (±2.6)	15.3 (±4)	.10	3 (±3.5)	0.53 (±1.6)	.02
*n° daily micturitions*	10.3 (±2.2)	10.8 (±2.4)	.53	11.5 (±1.3)	10 .9 (±2.1)	.44	1.23 (±1.7)	0.13 (±1)	.04

Mean pre-treatment VAS was comparable in the two groups (4.46 (2;10) for Group A and 4.86 (2;8) for Group B, p = 0.56). After treatments mean VAS was significantly lower in group B (4.2 (2;7) vs. 5.53 (4;7) in Group A, p = 0.04). VAS post treatment was increased in Group A and reduced in Group B: difference in post vs pre-treatment VAS was significantly lower in Group B (-0.66 (-4;1) vs. 1.06 (-4;3) in Group A, p = 0.0001).

Mean pre-treatment IPSS was comparable in both groups (13.93 (8;24) in Group A and 14.73 (9;22) in Group B, p = 0.6). After treatment, mean IPSS was slightly lower in group B (15.26 (11;21) vs. 17.46 (13;21) in Group A, even if the difference was not statistically significant p = 0.1). IPSS increase post treatment was significantly lower in Group B than in Group A (0.53 (-3;2) vs. 3.53 (-3;10) in Group A, p = 0.02).

Mean pre-treatment number of daily micturitions was comparable in the two groups (10.26 (7;15) for Group A and 10.8 (7;16) for Group B, p = 0.53). After treatment, we failed to find a significant difference in number of daily micturitions between the two groups (11.26 (9;13) in Group A and 10.73 (7;14) in Group B, p = 0.44). On the other hand, there was no increase in number of daily micturitions in Group B and a slight increase in Group A: this difference was significant (-0.066 (-2;1) for Group B and 1 (-2;5) for Group A, p = 0.04).

At 6 month follow-up 3/15 (21%) pts of Group A (including the patient who dropped out from the induction BCG cycle for severe LUTS) and 4/15 (26.7%) pts in Group B had a recurrence of BCa. None of them had BCa progression to muscle-invasive disease.

Several trials in the last two decades have investigated BCG efficacy in reducing the risk of recurrence and progression in intermediate/high risk BCa showing that BCG after TUR is superior to TUR alone or TUR and conventional chemotherapy [14,15]. Two meta-analyses have demonstrated that BCG therapy prevents, or at least delays, the risk of tumour progression [16,17].

Despite of its great efficacy, BCG therapy has potential local and systemic side effects that may either lead to treatment cessation in up to 30% of patients or lead to a delay or reduction in the number of instillations in 55–83% of patients [18].

The risk of increased toxicity during maintenance has been questioned. According to the results of a European Organization for Research and Treatment of Cancer (EORTC) phase 3 trial [18], local side effects of BCG do not increase during maintenance and systemic side effects are more frequent during the first 6 mo of treatment. However, a significant proportion of patients (84% [19], 67.3% [18]), 86% [20] in the three most representative series) failed to complete the 3-yr maintenance course for various reasons.

Several options have been proposed to decrease the occurrence of BCG side effects including the prophylactic administration of isoniazid or ofloxacin, modifications to the BCG treatment schedule and dose reductions. The concomitant administration of isoniazid, has not been found to decrease the incidence of BCG related side-effects [5]. In contrast, ofloxacin reduced the incidence of moderate to severe BCG related adverse events when given prophylactically in a randomized double blind trial in 115 patients [6,7]. However, further studies are required to confirm these initial findings and to ensure that there is no impairment of treatment efficacy. Martinez-Pineiro et al. investigated the BCG side effects rate using a reduced dose of BCG. They found significantly less toxicity on the reduced dose but recommended the use of a full dose in the treatment of high-risk patients [8]. In a second study in high-risk patients with T1G3 tumors and/or carcinoma in situ (CIS) they concluded that one-third dose was indeed as effective as full dose, but was associated with significantly less toxicity [21]. The final results of an EORTC-GU Cancers Group Randomized Study of Maintenance Bacillus Calmette-Guerin in Intermediate- and High-risk Ta, T1 Papillary Carcinoma of the Urinary Bladder showed no significant differences in toxicity between 1/3 dose and full dose (FD) BCG. 1/3 dose with 1 yr of maintenance was considered suboptimal compared with the standard FD during 3 yr [22].

Despite of this great number of papers on BCG toxicity in literature, only two papers investigated the possible use of HA for treatment of BCG induced cystitis [11,12] showing interesting results.

GAGs have been used extensively in BPS/IC with response rates between 30% and 80% described with intravesical administration of various substances such as HA, PPS, heparin; chondroitin sulfate, and DMSO [23,24]. The efficacy of HA is based on several mechanisms that aim on the urothelial function disorder present in BPS/IC: on one side, HA reinforces the urine-tissue barrier by integration in the GAG layer on the luminal surface and the base of urothelial cells; on the other side, unique anti-inflammatory mechanisms have been identified, like inhibition of leukocyte migration, adherence of immune complexes, and binding to specific receptors (I-CAM 1, CD 44) involved in the inflammatory process [25-27].

Moreover studies on rat models have shown that HA can inhibit bladder mast cell activation as well as the inflammatory mediator release of urinary histamine, rat mast cell protease-I and IL-6 [28] thus reducing their pro-inflammatory activity.

Because of GAGs documented anti-inflammatory and protective activity on the urothelium we considered the possible use of these devices on the treatment of BCG induced cystitis, even if one can argue that reducing BCG side-effects can even impair BCG efficacy.

A correlation between BCG side-effects and treatment efficacy has been reported by various authors, suggesting that local side-effects have a significantly longer time to first recurrence. However, patients with a better outcome remain on treatment for a longer period of time and receive more BCG, thus increasing their risk of developing side-effects. Neither local nor systemic BCG toxicity before 6 months was found to be a prognostic factor for subsequent recurrence. Thus, it is not possible to confirm that BCG toxicity is actually responsible for an improved outcome and a causal effect cannot be inferred from the data [29].

Our preliminary results show a possible role of HA in reducing BCG side-effects during the induction cycle when their frequency is higher than in the maintenance period [18]. As described in the Results and discussion section, VAS for bladder pain was significantly lower, at the end of the induction cycle, in Group B (patients treated with HA). It is important to underline that baseline VAS was relatively high because obtained in patients who recently underwent a TURBT. Interestingly, patients in Group B showed a reduction, whilst those in Group A presented an increase of VAS for pain. Indeed we failed to show between groups a statistically significant difference in IPSS and number of daily micturitions; nevertheless, the use of HA seems to have reduced the appearance of lower urinary tract symptoms in Group B, with an increase of IPSS of only 0.53 points vs. a significantly higher increase in group A (3.53). The same could be said about number of daily micturitions that remained almost unchanged in Group B whilst was increased after treatment in Group A. These results could indicate a protective function of HA on the urothelium of the bladder against the irritative activity of BCG.

One can argue that even ofloxacin when given prophylactically before each BCG administration [6,7] can achieve good results with a lower cost for the Health Care System. Actually the use of Ofloxacin has significantly decreased the incidence of class 2 or higher AEs, but has not improved class 1 AEs [6] while HA in our paper has demonstrated to significantly reduce class 1 AEs. Moreover it should be discussed if the prophylactic use of Ofloxacin for 9 weeks could increase bacterial resistance.

These very preliminary data seem to support a possible role of HA in reducing BCG local side effects. Nevertheless, our study has some limitations:

1. We have only a short follow up time;

2. It is a pilot study, with no power calculation;

3. Does not provide data on maintenance treatment;

4. Does not provide data on possible long term interactions of HA with BCG efficacy;

5. It remains to be seen whether the administration of HA after BCG is worth the benefit and increased costs.

The only outcome measures evaluated (VAS, IPSS, daily micturitions) may have not detected important efficacy end points, such as quality of life and relief of painful micturition.

For all these reasons, no definitive conclusions should be drawn. Nevertheless the data coming from this study should be used to design a randomized controlled study aimed to verify this hypothesis.

## Conclusions

This pilot study provides, for the first time to our knowledge, evidence of a possible reduction of BCG local adverse events by using a sequential administration of HA. Larger randomized controlled study, designed on the basis of this pilot study are needed to assess how clinically significant the association of HA to BCG could be. In other words, further studies will have to investigate if this association is worthy, having proven (statistically and clinically) significant reduction of local adverse events of BCG, possibly causing a reduction of the number of patients who have to suspend or discontinue the treatment with BCG, with a consequent reduction of its efficacy. Furthermore, a possible interaction of HA with BCG efficacy must be excluded before this association could become part of a standard care. In conclusion, this pilot study is only the first step, but further research is needed to investigate the exact potentialities and role of HA administered in patients treated by means of BCG for NMIBC.

## Abbreviations

HA: Hyaluronic acid; BCG: Bacillus Calmette-Guérin; NMIBC: Non-muscle invasive bladder cancer; VAS: Visual analog scale; IPSS: International Prostate Symptom Score; GAG: Glycosaminoglycan; BPS/IC: Bladder pain syndrome/interstitial cystitis; EORTC: European Organization for Research and Treatment of Cancer; TUR: Trans-urethral resection; BCa: Bladder cancer

## Competing interests

Enrico Finazzi-Agrò is consultant for Mylan Inc. Luca Topazio, Roberto Miano, Valentina Maurelli, Gabriele Gaziev, Valerio Iacovelli and Mauro Gacci declare that they have no conflict of interest.

## Authors' contributions

LT has been involved in drafting the manuscript. VI and GG have made substantial contributions to acquisition and analysis of data. VM has made substantial contributions to conception and design of the study. EFA, MG and RM have made substantial contributions to conception and design and interpretation of data; they have been involved in revising the manuscript critically for important intellectual content. All authors read and approved the final manuscript.

## Pre-publication history

The pre-publication history for this paper can be accessed here:

http://www.biomedcentral.com/1471-2490/14/64/prepub

## Supplementary Material

Additional file 1 A complete CONSORT Checklist. Click here for file
